# Supplementary material for: Exploring dairy heifers’ consistency in social motivation in the absence or presence of conspecifics
Source: PLoS One. 2025 Oct 29;20(10):e0334000. doi: 10.1371/journal.pone.0334000 (PMC12571274; doi:10.1371/journal.pone.0334000)
Supplement: S1 Appendix — (DOCX) [file pone.0334000.s001.docx]

**S1 Appendix. Sample size estimation.**

We derived our sample size from the average number of animals used in studies on dairy cattle personality assessment (n=10), resulting in 36 ± 16 animals (mean ± SD; range 17 - 56).
