# Supplementary material for: Exploring dairy heifers’ consistency in social motivation in the absence or presence of conspecifics
Source: PLoS One. 2025 Oct 29;20(10):e0334000. doi: 10.1371/journal.pone.0334000 (PMC12571274; doi:10.1371/journal.pone.0334000)
Supplement: S2 Appendix — (DOCX) [file pone.0334000.s002.docx]

**S2 Appendix. Randomization of animals and treatment groups.**

Animals were randomly assigned to a subgroup (three heifers) using Excel RANDBETWEEN(1:100) function to give each animal a random number. Animals were then sorted by random number in ascending order, and the first three animals were assigned to subgroup 1, the next three to subgroup 2 and so on. The same three animals that were tested during the distribution test were also used as test and companion animals in the trade-off test. We used the same randomization technique to assign treatment groups (i.e., social first, alone first in the trade-off test; start side for the distribution test) by giving each subgroup a random number.
