# Supplementary material for: Exploring dairy heifers’ consistency in social motivation in the absence or presence of conspecifics
Source: PLoS One. 2025 Oct 29;20(10):e0334000. doi: 10.1371/journal.pone.0334000 (PMC12571274; doi:10.1371/journal.pone.0334000)
Supplement: S3 Appendix — (DOCX) [file pone.0334000.s003.docx]

**S3 Appendix. Inter-observer reliability testing for novel arena and novel object test measures.**

Two independent observers assessed 20 % of the videos (11 novel arena test and 11 novel object test videos) for inter-observer reliability on the total duration of behaviors: novel arena test icc=0.93 (CI 0.89 – 0.96), novel object test icc=0.89 (0.84 – 0.93); intra-observer results for novel arena test: observer 1 icc=0.98 (CI 0.97 – 0.99), observer 2 icc=0.98 (0.97 – 0.99); intra-observer results for novel object test: observer 2 icc=0.97 (CI 0.95 – 0.99); observer 2 assessed all novel object videos).
