# Supplementary material for: Exploring dairy heifers’ consistency in social motivation in the absence or presence of conspecifics
Source: PLoS One. 2025 Oct 29;20(10):e0334000. doi: 10.1371/journal.pone.0334000 (PMC12571274; doi:10.1371/journal.pone.0334000)
Supplement: S4 Appendix — (DOCX) [file pone.0334000.s004.docx]

**S4 Appendix. Distribution test habituation.**

To habituate the animals to the distribution test arena, each group of heifers was exposed to a 20-minute habituation session daily for three consecutive days. In each session, heifers were first released into the arena and given 5 minutes to explore. Then, 150 g of grain was delivered into one of the two troughs (start side counterbalanced across the 12 subgroups) by a handler positioned outside the arena on the other side of the trough (not visible to the animals). The handler reached through gaps in the back wall of the trough to spread the feed evenly across the entire trough length using a 60 ml scoop. Simultaneously, a second handler shook a bucket filled with 500 g grain (only used as an acoustic signal; not delivered to the animals) for 10 seconds to signal the feed delivery and to encourage the animals to approach the trough. After 2 minutes (pilot trials showed that heifers consumed 150 g of grain within this time), another handler entered the arena and gently moved the animals to the second trough, visually separated and ~26 m away. As the heifers approached the other trough, the second handler shook the bucket again, and the first handler delivered grain into the second trough. This process of moving between the two feeding troughs was repeated 10 times in the session to facilitate habituation of moving back and forth between the two troughs. With increasing habituation, heifers started moving to the opposite trough voluntarily with the handler only entering the pen to move animals if they did not move after 2 minutes when all grain in the trough had been consumed. We did not apply a learning criterion for habituation as it was not our intention to train the heifers to associate specific cues (e.g., the sight of the handler approaching to move the animals) with switching between feeding sites. Instead, habituation sessions aimed to familiarize the heifers with feeding from both troughs so that the animals were comfortable moving freely between sides. If animals still needed encouragement to switch in the 3^rd^ habituation session (i.e., none of the three animals moved voluntarily during the last three two switches; this was the case for 1 subgroup in group 1, 3 subgroups in group 2, 2 subgroups in group 3), both troughs were filled with 150 g grain simultaneously at the end of the session and animals remained in the test pen until all three heifers had voluntarily visited both troughs.
