# Supplementary material for: Exploring dairy heifers’ consistency in social motivation in the absence or presence of conspecifics
Source: PLoS One. 2025 Oct 29;20(10):e0334000. doi: 10.1371/journal.pone.0334000 (PMC12571274; doi:10.1371/journal.pone.0334000)
Supplement: S5 Appendix — (DOCX) [file pone.0334000.s005.docx]

**S5 Appendix. Grain amount determination for the distribution test.**

We selected the grain quantity for the distribution test with the goal of encouraging continuous feeding at a low competition level while motivating heifers to move between troughs in order to access grain at an alternative location. This setup was intended to assess the trade-off between social interaction and feeding motivation. To determine an appropriate grain amount, we conducted pilot trials with three heifers (different from the test animals), measuring the time it took them to consume varying grain portions (250 g, 200 g, and 150 g). The 150 g portion was consistently depleted within approximately 2 minutes. This time interval enabled us to deliver grain up to 11 times within a 22-minute test period, which was the maximum available time for us to test each of the groups of three test heifers per day.
