# Supplementary material for: Exploring dairy heifers’ consistency in social motivation in the absence or presence of conspecifics
Source: PLoS One. 2025 Oct 29;20(10):e0334000. doi: 10.1371/journal.pone.0334000 (PMC12571274; doi:10.1371/journal.pone.0334000)
Supplement: S6 Appendix — (DOCX) [file pone.0334000.s006.docx]

**S6 Appendix. Companion vocalization analysis.**

A single observer recorded all vocalizations emitted by the companion animals during testing. Trade-off test durations varied depending on the test animals’ responses, which made it difficult to statistically test the effect of vocalizations on the test animals’ performance. However, because return latency was slower when tested in the social condition, we explored whether the number of calls from the companions influenced the test animals' motivation to return to zone 1. We hypothesized that hearing calls might have reassured the presence of conspecifics and therefore possibly reduced the test animals’ motivation to seek proximity to peers. We ran a linear mixed model with return latency as the dependent variable, the combined number of calls from the two companions, treatment (social, alone) and test repeat as the predictor variable and the subject as the random factor. We found no relationship between latency to return and companion calls (0.01 ± 0.01, R^2^=0.56, *p*=0.33).
