# Supplementary material for: Exploring dairy heifers’ consistency in social motivation in the absence or presence of conspecifics
Source: PLoS One. 2025 Oct 29;20(10):e0334000. doi: 10.1371/journal.pone.0334000 (PMC12571274; doi:10.1371/journal.pone.0334000)
Supplement: S1 Table — (DOCX) [file pone.0334000.s008.docx]

**S1 Table. Ethogram of behaviors assessed in the social isolation tests.** Animals were tested in three different tests: novel arena (NAT), novel object (NOT) and runway test (RWT).

| **Behavior**  **(measurement unit)** | **Description of measures** | **Test** |
| --- | --- | --- |
| Exploring  (% of time) | The animals’ muzzle or tongue is in contact or close distance (less than 5cm distance) with either walls or flooring substrate while moving or stationary. | NAT, NOT |
| Active  (% of time) | The animal is walking or running by moving at least one foot at a time of the ground. | NAT, NOT |
| Standing  (% of time) | The animal is standing still with all four feet on the ground for ≥2 s and not sniffing the ground. | NAT,  NOT |
| Vocalization  (frequency) | The animal emits calls (open- or closed-mouth calls) during testing. | NAT, NOT,  RWT |
| Latency to contact  novel object  (s) | The time passed between the animal entering the test pen with all four legs until its muzzle is in contact with the object. | NOT |
| In contact with object  (% of time) | The animals’ muzzle/tongue is in contact with object (muzzle within 5 cm distance) including head in contact with the object (‘butting’). | NOT |
| Latency to reestablish proximity to peers  (s) | The time passed from the moment the holding pen opened until the test animals is within 5 m proximity to its peers. | RWT |
