# Supplementary material for: Exploring dairy heifers’ consistency in social motivation in the absence or presence of conspecifics
Source: PLoS One. 2025 Oct 29;20(10):e0334000. doi: 10.1371/journal.pone.0334000 (PMC12571274; doi:10.1371/journal.pone.0334000)
Supplement: Attachment — Submitted filename: Response to Reviewers - Social Heifers - Kappel et al..docx [file pone.0334000.s010.docx]

Dear reviewers,

Thank you very much for taking the time to review our manuscript and provide comments to help us improve our article. We address specific comments below:

Reviewer #1:

Line 18 - Area should be arena

Corrected.

Line 50 - There is a possibility that sociability may also be separation distress. Jack Panksepp calls this PANIC

Panksepp, J. (2011) The basic emotional circuits in mammalian brains: Do animals have affective lives? Neuroscience and Biobehavioral Reviews, 35(2011), 1791-1804

Thank you. We agree with the reviewer and have added the suggested reference and the text “or as a signal of separation distress” in L 51.

Line 54 - I agree that social isolation may reflect more than on personality trait.

Thank you.

Line 117 - State the breed of the heifers

Added in L 118.

Discussion - You need to think about the differences between birds and sheep and your dairy heifers. Both birds (27) and grazing sheep (22) have greater freedom to move about in the space where they forage. It is also possible that foraging birds and grazing sheep had greater hunger motivation than your very well fed heifers. The birds and sheep are also not in a situation where they could be eating from the same feed. Could the birds and sheep living in a more extensive environment had an effect on the results?

We agree that free-ranging animals might differ in their willingness to disperse from peers in exchange for food, especially if feed is available less consistently. We specified that the discussed example of sheep behaviour was done in free-ranging animals by added “free-ranging” before sheep in L 570.

We added “Finally, we encourage continued research to explore the conditions under which the IFD model applies to confined social animals” in L 596 – 597 to clarify that the suitability of IFD as a framework for measuring social motivation in confined animals needs further investigation.

This reference may be helpful. It shows that two scores that are often being used for measuring cattle temperament may be measuring different traits.

Bruno, K. et al. (2016) Relationship between quantitative measures of temperament and other observed behaviors in growing cattle, Applied Animal Behavioral Science, 199:56-66.

Thank you for your suggestion. After reviewing the article, we have decided not to include this reference, as we regard personality and temperament as distinct concepts. Given the breadth of information already presented to the reader, we believe that introducing an additional concept may lead to unnecessary complexity.

Reviewer #2:

This is an excellent manuscript that has presented a complex range of results from varying behavioural tests and the relationships between the test outcomes.
I struggled to find ways to improve the text.

Thank you.

Lines 39-40 reads a bit like a repeat of the previous text.

Corrected in L 39. We replaced the repetition of “reunited with” with “return to its group”.

Line 65: should this be 'in exchange for feed;'?

Corrected in L 66.

Line 195: delete double use of 'contact'

Corrected in L 196.

Lines 326-328: can you state how many animals this equates to for not leaving zone one.

We state the number of animals leaving and not leaving zone in the results section, L 401 and 402.
